# Editorial: RNA binding proteins in neuroscience

**DOI:** 10.3389/fnmol.2023.1340721

**Published:** 2023-12-05

**Authors:** Anca F. Savulescu, Epaminondas Doxakis

**Affiliations:** ^1^Division of Chemical, Systems and Synthetic Biology, Institute for Infectious Disease and Molecular Medicine, Faculty of Health Sciences, University of Cape Town, Cape Town, South Africa; ^2^Center of Basic Research, Biomedical Research Foundation, Academy of Athens, Athens, Greece

**Keywords:** RNA, RNA binding protein, neurodegenarative disease, neuronal processes, neuroscience

Unraveling the complexities of brain function relies on the precise coordination of gene expression in different neural subtypes in a spatiotemporal manner. RNA Binding Proteins (RBPs) play a central role in gene expression, acting as versatile modulators in processes ranging from alternative splicing to mRNA transport and translation. Despite progress in understanding the diverse roles of RBPs, most details of their context-specific functions remain enigmatic. This Research Topic uncovers new evidence regarding the roles of RBPs in neuronal processes and their implication for neurodegenerative diseases. The six manuscripts in this Research Topic unravel the roles of RBPs in diverse scenarios, including the regulatory pathway of melatonin in traumatic brain injury, the dysregulation of lipid metabolism in frontotemporal lobar degeneration, the connection between miRNAs, their target mRNAs, and their encoded proteins, and the changes in gene expression after exposure to ethanol. As we explore the intricate details of RBPs at the molecular level, these studies together highlight the crucial role of RNA-protein interactions as indispensable coordinators shaping the complex landscape of neurological processes and dysfunction.

Fu et al. investigated the regulatory pathway of melatonin, a neuroendocrine hormone, in a mouse model of traumatic brain injury (TBI) using transcriptomics and bioinformatics analysis. This study identified 259 differentially expressed RNAs between mice treated with melatonin and untreated mice, encompassing mRNAs, lncRNAs, miRNAs, and circRNAs. To delineate the biological functions of lncRNAs in melatonin-treated TBI mice, the authors employed StarBase (Li et al., 2014) to predict the RBP targets of these lncRNAs (Fu et al.). Notably, lncRmst interacted with up to fifteen RBPs, while the RBP FUS correlated with ten differentially expressed lncRNAs (Fu et al.). Interestingly, compared to control, eight RBPs were jointly regulated by higher and lower expressed lncRNAs in melatonin-treated mice (Fu et al.). Kyoto Encyclopedia of Genes and Genomes (KEGG) analysis, focusing on target genes of RBPs predicted to bind lncRNAs, highlighted enriched pathways such as axon guidance, glutamatergic synapse, adherens junction, hippo signaling pathway, and synaptic vesicle cycle (Fu et al.). Several of these cellular pathways have been previously linked to melatonin function in other diseases (Yuan et al., 2011; Evely et al., 2016; Stazi et al., 2021). Gene Ontology (GO) analysis revealed enrichment in cellular component organization, nervous system development, and neurogenesis (Fu et al.). This data offers fresh insights into the effects of melatonin treatment after TBI, emphasizing the role of RNA-protein interactions as potential key players (Fu et al.).

The ATP-binding cassette subfamily A (ABCA) transporters constitute a family of proteins responsible for regulating cellular lipid transport and have been implicated in various neurodegenerative diseases. However, the roles of ABCA transporters in frontotemporal lobar degeneration with TDP-43 inclusions (FTLD-TDP), a prevalent form of younger-onset dementia, remain unclear. TDP-43, encoded by the TARDP gene, is an RBP associated with RNA processing and nucleocytoplasmic transport (Ravanidis and Doxakis, 2020). TDP-43 pathology has been linked to several neurodegenerative diseases (Ravanidis and Doxakis, 2020). In a study by Katzeff et al. the expression of 13 ABCA transporters was examined in five key brain regions in individuals with FTLD-TDP and controls. The results revealed that seven ABCA transporters (2–4, 7, 9, 10, and 13) exhibited differential expression in FTLD-TDP, and this occurred in a region-specific manner. Moreover, the expression of several ABCA transporters showed correlation or anticorrelation with neural subtype markers such as MAP2 (7), TMEM119 (4, 8, 9, 10, 13), TPPP (4), and GFAP (7, 8) as well as TDP-43 (4, 10, 13) in the different brain regions (Katzeff et al.). These findings suggest dysregulation of lipid metabolism in FTLD-TDP, particularly associated with neuroinflammatory microglia marked by TMEM119 (Katzeff et al.).

In another study highlighted in this Research Topic, Krishnan et al. explored a distinct RBP. Their investigation focused on unraveling the impact of ethanol (EtOH) and Poly (ADP-ribose) polymerase (PARP) inhibition on ribosome-bound RNA transcripts in prefrontal cortical (PFC) pyramidal neurons. PARP, recognized as a multifunctional RBP, predominantly binds nascent transcripts, influencing the rates of various competing RNA processing steps (Melikishvili et al., 2017). Mice were subjected to twice-daily EtOH treatments for four consecutive days, followed by administering the PARP inhibitor ABT-888 (Krishnan et al.). RNA-seq analysis was conducted on ribosome-engaged RNA from CaMKIIα neurons, in mice expressing the EGFP-tagged Rpl110a ribosomal subunit in pyramidal cells, and genomically expressed total RNA from whole tissue (Krishnan et al.). The study revealed that EtOH treatment induced alterations in mRNA transcripts in ribosomal engagement in pyramidal cells and total tissue, and this effect could be reversed by 82–83% with the administration of the PARP inhibitor (Krishnan et al.). Intriguingly, the insulin receptor signaling pathway emerged as highly enriched in transcripts affected by EtOH treatment and restored by PARP inhibition (Krishnan et al.). In essence, this data explains the consequences of EtOH on ribosome-engaged transcripts and proposes a role for the RBP PARP in regulating the effects of EtOH (Krishnan et al.).

An additional study within this topic focused on the dynamics of miRNAs and their mRNA targets in the cortex and hippocampus across different stages of mouse development, spanning early, mid, and late adolescence to adulthood (Thomas et al.). In this study RNA sequencing was used to uncover that ~25% of miRNAs' 3'-ends undergo shortening with age, attributed to increased 3' trimming and reduced U-tailing (Thomas et al.). This phenomenon was observed in other datasets from both mouse and human miRNA-seq (Thomas et al.). Compared to miRNAs with stable 3' ends, those with shortened ends displayed stronger positive correlations with mRNA targets that increase with age and stronger negative correlations with mRNA targets that decrease with age (Thomas et al.). This indicates the conservation of 3' end miRNA shortening during postnatal brain maturation (Thomas et al.). Furthermore, the researchers conducted quantitative proteomics using tandem mass-tag mass spectrometry (TMT-MS) on the same cortical samples used for RNA-sequencing (Thomas et al.). The TMT-MS data revealed age-related protein decreases associated with cytosolic and mitochondrial mRNA translation, mRNA splicing, RNA binding, and canonical miRNA biogenesis (Thomas et al.). At the same time, proteins linked to myelination, synaptic activity, cellular respiration, and ribonucleotide synthesis increased with age (Thomas et al.). The study then explored the relationship between miRNAs, their mRNA targets, and the encoded proteins, showing that miRNA levels poorly correlated with their mRNA targets, but exhibited stronger correlations with the proteins encoded by these mRNA targets (Thomas et al.). In conclusion, the findings suggest substantial alterations in RNA synthesis, function, and stability pathways, underscoring the growing significance of post-transcriptional regulation in gene expression during adolescent and early adult brain maturation (Thomas et al.).

In a mini review, Yang et al. explore the link between deficits in axonal transport within neurons and the aberrations observed in the accumulation of pathogenic proteins and subcellular localization of organelles, hallmark features of various neurodegenerative diseases. These transport deficiencies involve changes in the expression levels and post-translational modifications of motor proteins, destabilization of microtubules, and a decrease in the binding between cargo and motor proteins. These issues contribute to the early stages of neurodegenerative diseases by compromising axonal integrity. The authors compile a list of proteins with mutations that impact axonal transport, linking them to neurodegenerative conditions such as Alzheimer's disease, frontotemporal dementia (FTD), amyotrophic lateral sclerosis (ALS), and others (Yang et al.). Among these mutant proteins are the well-known RBP TDP-43, the kinesin motor protein KIF5A, which binds RNAs, including those encoding RBPs, affecting mRNA processing (Baron et al., 2022), and Huntingtin (HTT) mRNA and protein, implicated in disrupting RNA metabolism by recruiting RBPs (Schilling et al., 2019) and possessing RNA binding capacity (Culver et al., 2016; Yang et al.). Future studies will uncover more about axonal transport defects, the role of RBPs, and their potential as therapeutic targets in neurodegenerative diseases (Yang et al.).

Naskar et al. provide a thorough exploration of the involvement of RBPs in neurodegenerative diseases, with a specific focus on ALS/FTD. The review explores the intricate process of liquid-liquid phase separation of RBPs associated with these diseases, highlighting disruptions in phase transitions. This phenomenon involves the dynamic formation of biomolecular condensates comprising ribonucleoprotein (RNP) granules that assemble into functional compartments and higher-order structures within cells. These condensates are pivotal in RNA processing, transport, stability, and translation (Banani et al., 2017; Yang et al., 2020; Naskar et al.). The RNP granules, including stress granules and neuronal transport granules, are enriched in RBPs, such as FUS, TDP-43, and hnRNPA1 (Naskar et al.). Mutations and aberrations in RBPs within RNP granules disrupt interactions with RNA transcripts and other RBPs, leading to disturbances in phase transitions (Naskar et al.). Consequently, various functions of these granules, including stability, transport, and subcellular localization of mRNA transcripts, are affected, resulting in disruptions in broader neuronal processes such as local translation, axonal integrity, and synaptic function (Naskar et al.). This disruption contributes to the formation of pathological aggregates associated with several neurodegenerative diseases, including Alzheimer's disease, FTD, and ALS (Naskar et al.). The review further explores the molecular mechanisms underlying the abnormal assembly of RNP condensates in neurodegenerative diseases, discusses cellular mechanisms to overcome aberrant phase separation, and explores potential therapeutic approaches (Naskar et al.).

In conclusion, the growing body of evidence highlights the engagement of RBPs in various neuronal cellular processes and their link to mutations and abnormalities in RNA-protein interactions associated with many neurodegenerative diseases. To comprehend the pathology of these diseases and potentially identify novel therapeutic targets, it is crucial to gain insights into the molecular mechanisms underlying the roles of RBPs in these processes. Future studies will play a pivotal role in refining and substantiating the current evidence, contributing to the advancement of knowledge in this field.

## Author contributions

AS: Conceptualization, Writing – original draft, Writing – review & editing. ED: Conceptualization, Writing – original draft, Writing – review & editing.
